# Riluzole and Edavarone: The Hope Against Amyotrophic Lateral Sclerosis

**DOI:** 10.7759/cureus.30035

**Published:** 2022-10-07

**Authors:** Aditi V Rokade, Pallavi Yelne, Anamika Giri

**Affiliations:** 1 Department of Medicine, Jawaharlal Nehru Medical College, Datta Meghe Institute of Medical Sciences, Wardha, IND

**Keywords:** hyperreflexia, riluzole, hyporeflexia, oxidative stress, sod1 gene, pathogenesis, amyotrophic lateral sclerosis, upper motor neurons, lower motor neurons, motor neuron disease

## Abstract

Amyotrophic lateral sclerosis (ALS) is one of the most frequent motor neuron illnesses. Motor neuron illnesses are various disorders that include upper and lower motor neuron abnormalities. Amyotrophic lateral sclerosis accounts for roughly 80% of motor neuron disorders. ALS is a fatal motor neuron disease that involves the loss of motor neurons in the spinal cord and brain, resulting in gliosis and muscle weakening and wasting in the upper, lower, and respiratory muscles, reducing life expectancy to 2-5 years from the onset of symptoms. Up until now, oral riluzole, a glutamatergic neurotransmitter inhibitor, has been used to manage ALS, the only drug for the management of ALS that has been approved by the United States (US) Food and Drug Administration (FDA). In recent studies, edaravone has been used through intravenous mode to halt the progression of ALS. We conducted a systematic search on PubMed; we selected Google Scholar, PubMed, websites regarding ALS, WebMD, Researchgate, als.org, consultant360, and the relevant articles for the review. It shows us riluzole and edaravone's efficacy for managing A.L.S. and how it can increase the life span of the patients.

## Introduction and background

Amyotrophic lateral sclerosis (ALS) is the most common motor neuron disease, accounting for approximately 80% of all motor neuron illnesses. It causes both upper and lower motor neuron lesions, which have symptoms such as weakness in the upper and lower limbs, twitching, hypertonia, and hyperreflexia. It also includes symptoms that progress to the respiratory system, such as breathlessness on minimal exertion, even when lying flat. As a result, patients develop aspiration pneumonia and respiratory inadequacy. These are terminal events, and it also includes symptoms that progress to the digestive system, including breathlessness on minimal exertion. This progressive neurodegenerative condition, i.e., ALS, destroys nerve cells in the brain and spinal cord [1]. About 80% of the patients have swallowing issues, which might negatively impact metabolism. The body begins to break down muscles for energy, which can cause weight loss in ALS patients [2]. According to epidemiological studies of generic ALS, the greatest prevalence is seen in individuals in their 70s and 80s, and the median survival period is two to four years [3]. Males are more likely than females to develop ALS. We have the portrayal of ALS dated back to 1824 by Charles Bell, and Jean-Martin Charcot described the symptoms and pathophysiology in 1869. It became a known public disease when legendary footballer player from the US, Lou Gehrig, died of ALS in 1941. We have the more recent news of ALS of Stephen Hawking, who passed away on March 14 2018. 

In the 160 years of the disease's existence, clinical studies have been carried out in numerous academic institutions and businesses worldwide without producing a curative therapy. Pharmaceutical companies have tested several animal models of sickness over the past few decades to find a cure and delay the disease's course; still, they have not been successful in determining whether the treatments work as intended [4].

Pathogenesis

ALS is a common name for motor neuron disease, a paralytic disorder caused by degeneration of the motor neurons that causes muscle weakness and leaves the patient with 2-5 years of life [5]. Upper motor neurons (UMNs) and lower motor neurons (LMNs) are the two types of motor neurons. Upper motor neurons are the first to transport movement impulses from the cerebral cortex, brainstem, and spinal cord. They are further divided into the corticospinal tract and corticobulbar tracts [5]. Damage to the UMN leads to a set of characteristic symptoms: weakness, spasticity, and hyperreflexia. Lower motor neurons are present in the anterior horn of the spinal cord. They are the efferent group of neurons of the peripheral nervous system, which connects the central nervous system with the muscles supplied. The lower motor neuron brings the entire function of the central nervous system [6]. The leading cause of ALS is not yet known. The ALS Association states that there are two forms of ALS: the most prevalent variety, which is sporadic and can afflict anyone, and the inherited type, which is familial [7].

**Figure 1:**
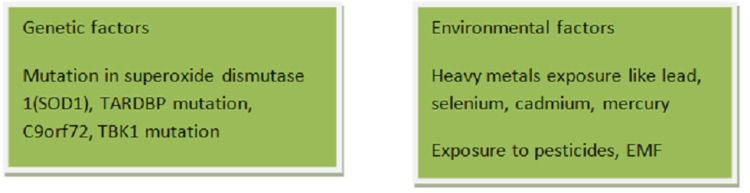
Risk factors predisposing to amyotrophic lateral sclerosis TARDBP: Transactive response DNA binding protein, C9orf72: chromosome 9 open reading frame 72, TBK: tank binding kinase, EMF: electromagnetic field Image credit: Author Aditi Rokade

SOD1 is a copper and zinc-containing superoxide dismutase. It is a highly abundant copper enzyme which contains as much as 1% of the total cell proteins and is an antioxidant enzyme that guards the cells against the toxicity of oxygen-free radicals. Its responsibility is to combat free radicals. In other words, some familial instances are caused by mutations in the cytosolic superoxide dismutase (SOD1) gene [8]. It is also claimed to be a cytosolic and mitochondrial enzyme. It results in the superoxide being changed into molecular oxygen and hydrogen peroxide. The familial mutation is the most common cause of ALS joint involvement in familial ALS. The risk factors predisposing to ALS are given in Figure 1.

**Figure 2:**
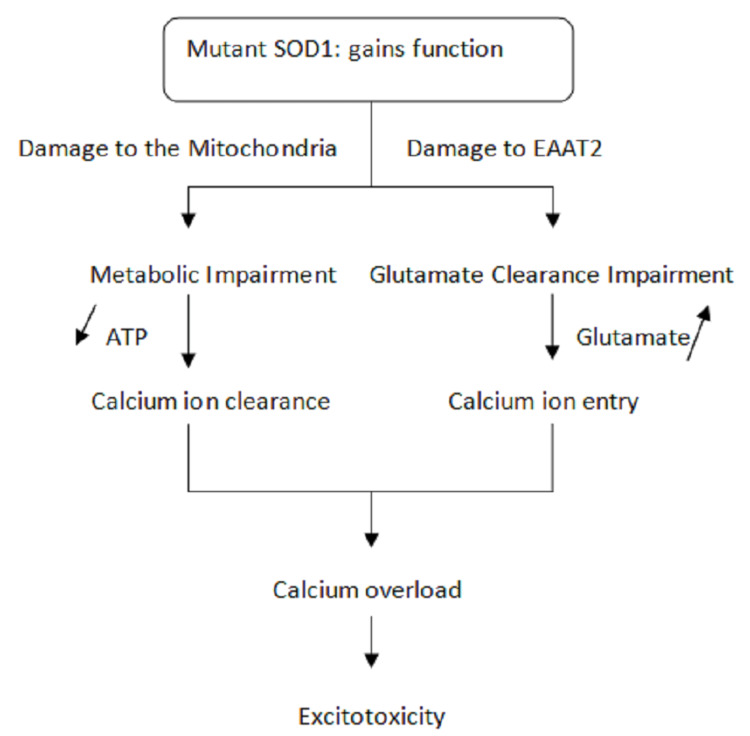
Pathogenesis of amyotrophic lateral sclerosis SOD1: superoxide dismutase 1, ATP: Adenosine Triphosphate, EAAT2: Excitatory amino acid transporter 2 Image credit: Author Aditi Rokade

**Figure 3:**
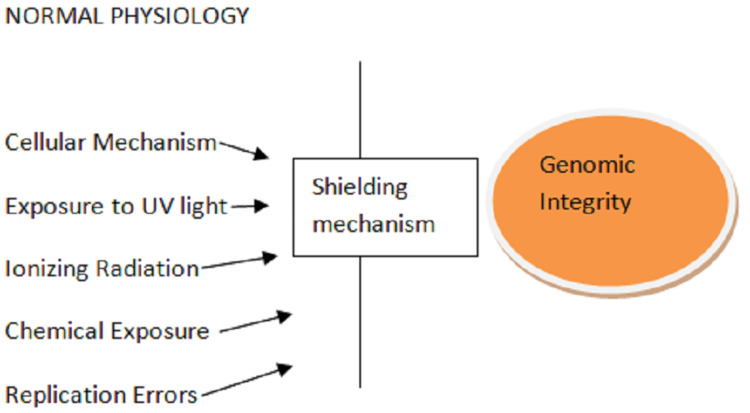
Normal physiology in a genome against mutants UV: ultraviolet radiation Image credit: Author Aditi Rokade

**Figure 4:**
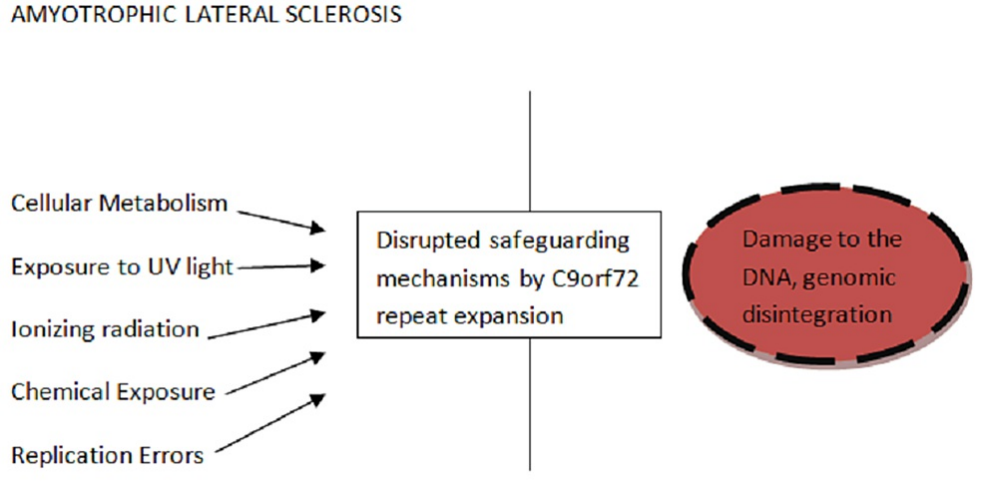
Loss of genome integrity in amyotrophic lateral sclerosisbecause of mutants like C9orf72 C9orf72: chromosome 9 open frame reading 72, DNA: deoxyribonucleic acid, UV: ultraviolet Image credit: Author Aditi Rokade

Alsin (ALS2) is a guanine nucleotide exchange factor required for proper cytoskeletal dynamics. It also includes the misfolded mutant of the SOD1, which stimulates microglia to produce extracellular superoxide. The environmental factors and the mutation are known to cause ALS. Figure 2 shows the pathogenesis of ALS. Figure 3 shows the maintenance of genome integrity by the shielding mechanisms. Figure 4 shows the disintegration of the genomic integrity because of the mutants.

Alterations in protein trafficking, glutamate excitotoxicity or neurotoxicity, aberrant accumulations of neurofilaments, and altered neurotropism are all plausible causes of sporadic ALS. One more mutation was found in the gene GGGGCC. In these individuals, there are multiple repetitions of the chromosome 9 open reading frame 72 (C9orf72) in the ranges of hundreds and thousands, developing ALS as compared to ordinary individuals with only a few proteins in their genes. Given the rarity of some particular mutations, it can be challenging to define genotype-phenotype connections. It has been shown that patients with SOD1 mutations typically experience lower limb onset [9], while those with transactive response DNA binding protein (TARDP) mutations typically experience upper limb onset. Patients with fused in sarcoma (FUS). mutations experience disease onset at a younger age and live shorter lives [9]. The genes involved in ALS and their loci are given in Table 1. 

**Table 1:** Genes that are mutated in amyotrophic lateral sclerosis ALS: amyotrophic lateral sclerosis, SOD: superoxide dismutase, SETX: senataxin, dHMN; distal hereditary motor neuropathies, ANG: angiogenin, PMA: progressive muscular atrophy, FTD: frontotemporal dementia, PBP: progressive bulbar palsy, OPTN: optineurin, TARDBP: transactive response DNA binding protein, ATXN2: ataxin-2, VAPB: VAMP Associated Protein B and C, FIG: Fused in Glioblastoma, PLS: Primary Lateral Sclerosis, SPG: spastic paraplegia, VCP: Valocin Containing Protein, Arg487: Arginase487, PDB: Paget's disease of bone, UBQLN2: ubiquilin-2, AD: autosomal dominant, AR: autosomal recessive

Locus	Gene	Inheritance	Phenotype
ALS one	SOD1	AD, AR	ALS, PMA
ALS two	ALS2	AR	Juvenile ALS, PMA
ALS three	Not defined	AD	ALS
ALS four	SETX	AD	ALS, dHMN
ALS five	SPG11	AR	Juvenile ALS
ALS six	FUS	AD, AR, De Novo	ALS, ALS-FTD
ALS seven	Not defined	AD	ALS
ALS eight	VAP8	AD	ALS, PMA
ALS nine	ANG	AD	ALS, ALS-FTD, PBP
ALS ten	TARDBP	AD	ALS, ALS-FTD
ALS eleven	FIG4	AD	ALS, PLS
ALS twelve	OPTN	AD, AR	ALS
ALS thirteen	ATXN2 C9orf72	AD AD	ALS ALS, FTD, ALS-FTD
ALS fourteen	VCP Arg487	AD, AR	ALS, PDB
ALS fifteen	UBQLN2	AD	ALS

The genes implicated in causation, modification of risk, or progression of ALS are SOD1, ALS2, SETX, SPGI1, FUS, VAPB, ANG, TARDBP, FIG4, OPTN, ATXN2, C9orf72 [10] and VCP, Arg487, and UBQLN2. According to recent research, motor neuron degeneration begins distally from the synaptic region [11]. The increased oxidative stress is the leading cause of ALS. 

Symptoms

It is a motor neuron illness that encompasses both UMN and LMN symptoms. Common examples of UMN symptoms include hyperreflexia, spasticity, fasciculations, and positive Babinski reflex. LMN signs include muscle wasting and twitching of the muscle involuntarily [12]. In early symptoms of ALS, the patients complain of difficulty in breathing with exertion and frequent sighs at rest. With the progression of the disease, the patients have dyspnea at rest. The initial symptoms include progressive muscle weakness and proximal symptoms, such as difficulty in raising hands. Because of atrophy of the tongue, the patient finds it difficult to swallow, which harms the metabolism and the patient experiences weight loss [2]. The muscles controlling speech and swallowing and those in the hands, arms, legs, and feet are also affected by symptoms. Additionally, patients need ventilatory support to breathe because of the involvement of the respiratory muscles. Muscles that control speech and swallowing and those in the hands, arms, legs, and feet are also affected [13].

The symptoms of ALS are further classified by body segments, which include the bulbar region (jaw, face, palate, and tongue), cervical region (neck, arm, hand and diaphragm), lumbosacral thoracic region (back and abdomen), and leg and foot. One of the typical symptoms is the cramping of the muscles. Due to tendon imbalance and consequent joint contractures, the stable condition can cause foot and hand abnormalities. Most ALS patients pass away while asleep, and the cause of death in this disease is typically an accumulation of carbon dioxide in the body.

Diagnosis

Neurologists should make a diagnosis, and various modalities have been found to make the diagnosis.

Electromyography (EMG)

EMG, also called needle EMG, is the next step when ALS is suspected. An electrode is put into each arm and leg, one proximal and one distal, to capture the electrical activity of the muscle, both at rest and during contraction [14]. An aberrant EMG is more common in ALS patients when there has been a significant loss of LMNs. We observe anomalous EMG signals even when the muscle is at rest [14]. Nerve conduction studies (NCS) determine if the motor neurons' signal strength is sufficient to permit muscle action.

D.N.A. analysis

This investigation may be recommended if familial ALS is suspected. Muscle biopsies can be performed to rule out specific muscle ailments.

Magnetic resonance imaging

An MRI allows imaging of the brain and spinal cord to rule out other disorders such as brain tumours and spinal cord abnormalities. A lumbar puncture, blood, and urine test are all performed [15]. If ALS is suspected, electromyography, also known as an EMG, is routinely performed. This test looks at the signals that pass between neurons and muscles and the electrical activity forces inside muscles to see if there is a pattern related to ALS. If this is the case, additional tests are almost certainly requested. Electromyography (EMG) patterns in ALS are caused by acute and chronic denervation and reinnervation of the affected muscles. According to some research, muscle ultrasonography can detect fasciculations that can aid in diagnosing ALS [16].

Blood tests

They are also carried out to rule out illnesses that resemble ALS. Blood tests are standard laboratory procedures for persons suspected of having ALS and patients who often do not exhibit any blood abnormalities. Blood tests are performed to rule out the presence of any other diseases that can be confused for ALS. For instance, a blood test can evaluate your hormone levels, blood cell count, liver function, and kidney function to see whether you have any illnesses [17].

Treatment modalities

There is no known cure for ALS. However, medical professionals have remedies and therapies that can make symptoms sluggish or lessen them. To understand the disease's causes and potential novel treatments, researchers are still researching ALS. Two drugs have been shown to reduce the severity of ALS and extend the lives of persons with the condition. They can delay the need for mechanical assistance with breathing, but they cannot reverse the harm already done [18].

Riluzole

Benzothiazole protects neurons from toxic damage in vitro and in vivo by blocking post-synaptic glutamate receptors, decreasing glutamate release, and inactivating voltage-dependent sodium channels. It has been demonstrated that riluzole inhibits presynaptic voltage-gated Na+ channels, reducing presynaptic glutamate release. Presynaptic voltage-gated calcium (Ca2+) channel inhibition may factor in the reduced glutamate release [2]. Riluzole was licenced in the US for usage in 1995 to treat amyotrophic lateral sclerosis. Under the trade name Rilutek, riluzole 50 mg pills are offered. At every 12-hour, a 50 mg maintenance dose is given as usual. The most typical adverse reactions are weakness, dizziness, nausea, diarrhoea, and cough [19]. Other side effects include that when combined with some medications (including Advil) for infections, birth control, high cholesterol, seizures, pain, or arthritis, riluzole might cause liver damage [20]. Riluzole may interact with other medications, including prescription and over-the-counter (OTC) drugs, vitamins, and herbal items.

Edaravone 

Edaravone is a free radical that is intended to reduce the effects of oxidative stress and has been linked to the development of ALS. Free radicals are very reactive. Unpaired electrons and oxidative stress disrupt the body's ability to detoxify or prevent their negative consequences. Oxidative stress biomarkers are frequently higher in ALS patients [21].

## Review

Objective

This study investigates the therapeutic effect and efficacy of riluzole and edaravone, the only two medications approved to treat ALS.

Methodology

**Figure 5:**
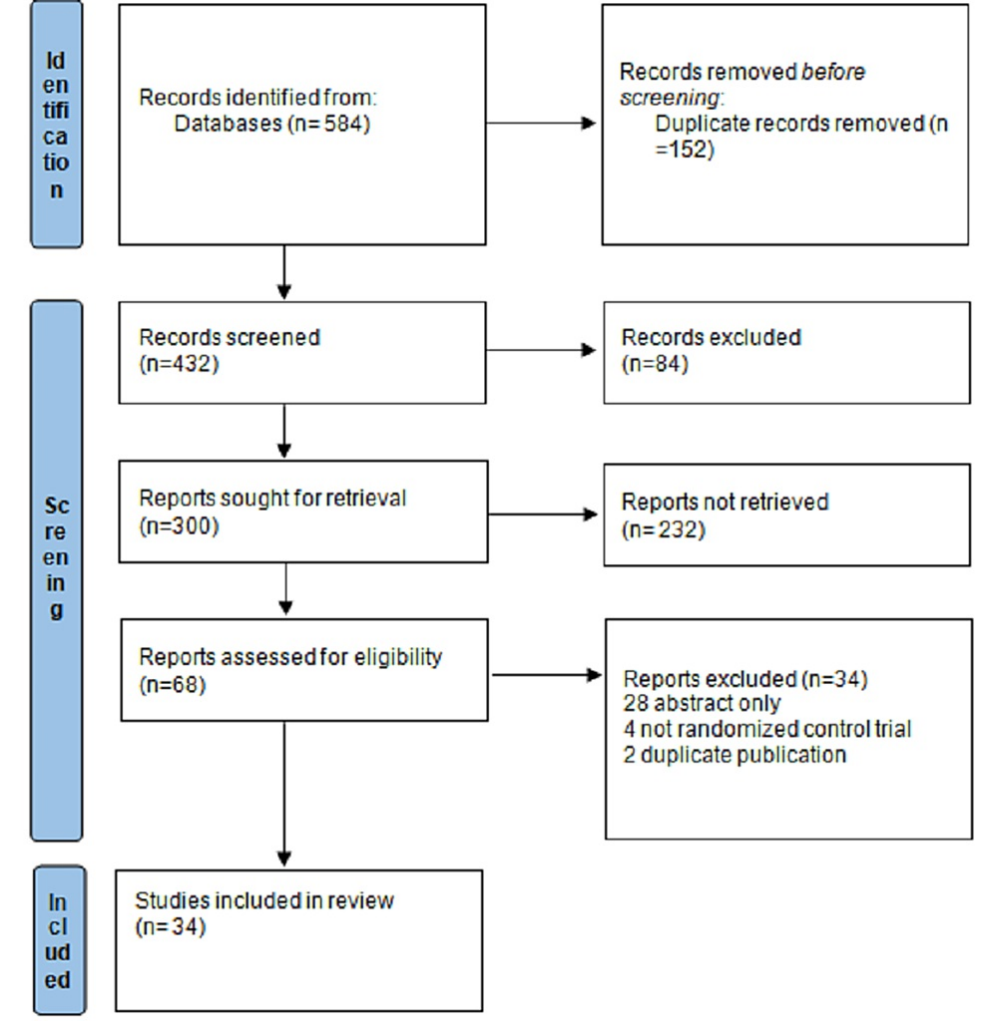
Preferred Reporting Items for Systematic Reviews and Meta-Analyses 2020 flow diagram for reviews which included searches of databases n: number Image credit: Author Aditi Rokade

We searched for studies evaluating the efficacy of edaravone and riluzole in ALS. For the introduction, we searched for papers on the mechanisms of action of the two medications. Using the medical subject heading (MeSH) term riluzole with the subheading pharmacology, we found 38 reviews. The search was narrowed down by including "A.L.S.", and we obtained 23 studies. We selected the article with the title "mechanism of action", "riluzole", and "amyotrophic lateral sclerosis". After reading the abstracts of the nine reviews that used the terms "Edaravone" and "A.L.S.", we chose one. Additionally, we looked up reviews in comparison between edaravone and riluzole, UMN and LMN pathology, genes mutated in ALS, clinical features and treatment modalities for ALS and they are given in Table 2. Figure 5 shows the flow chart involving the database searches and the reviews included in the article.

**Table 2:** Search items for amyotrophic lateral sclerosis A.L.S.: amyotrophic lateral sclerosis

Date	Search items	Platform	Reference no.
180722	A comparison between edaravone and riluzole; indication and efficacy	Google Scholar	[2]
180722	Upper motor neuron and lower motor neuron pathology	ScienceDirect	[3] [4]
190722	Genes mutated in A.L.S.	researchgate	[6]
190722	Clinical features of A.L.S.	1library.net	[8]
200722	Symptoms and diagnosis of A.L.S.	PubMed	[9][10]
210722	Treatment modalities	WebMD, als.org	[11] [12] [13]

Riluzole and edaravone: a tale of two amyotrophic lateral sclerosis drugs

Patients who participated in these trials had an initial forced vital capacity (FVC) of less than 60% and had had ALS for less than five years. The first trial followed 155 ALS patients from France and Belgium for at least 13 months after they received either 100 mg/day of riluzole or a placebo. The second trial included 959 people with ALS from North America and Europe. Patients were followed for at least 12 and 18 months after receiving riluzole at doses of 50, 100, or 200 mg/day or a placebo.

Sixty-nine individuals were given 60 mg of edaravone intravenously, while they gave 68 a saline placebo injection. A total of 24 weeks of treatment was done, including two weeks of edaravone or placebo and two weeks of no treatment (4 weeks = one cycle). For the first two weeks of cycle 1, one dosage of edaravone or placebo was administered daily. In cycles 2-6, edaravone or placebo was issued on 10 of the 14 days. The patient had to have received at least one treatment infusion, undergone at least one post-baseline evaluation, and finished at least 12 of the 24 weeks in order to be eligible for the primary outcome examination, which measured the change in the ALS Functional Rating Scale (ALSFRS-R) score from baseline to 24 weeks [4].

In the study, 876 riluzole-treated and 406 placebo-treated patients made up the overall population in the three trials looking at tracheostomy-free survival. The three studies had excellent methodological quality and were easily comparable, even though one involved older patients with more severe forms of amyotrophic lateral sclerosis.

Riluzole

In the first two trials, a benefit was seen for the same group of patients using riluzole 100 mg daily. Secondary survival studies at multiple time points revealed that riluzole 100 mg provided a substantial survival advantage at six, nine, twelve, and fifteen months, but not at three or eighteen months. Although muscle strength was unaltered, bulbar and limb function improved marginally. There were no statistics on life satisfaction. However, patients using riluzole had significantly more extended periods, making their health minimally impaired in comparison to patients taking a placebo [22]. Riluzole's effectiveness has been studied in observational population-based studies with a longer follow-up. However, the results are mixed, and it is still unknown whether the drug's effects are only shown in individuals at the initial stage of the disease or in specific patient subgroups [23].

According to one study, those with ALS who began long-term riluzole treatment in the early stages of the disease stayed for a longer period of time than people who started treatment in the later stages. According to another study, patients treated with riluzole for 90 per cent or more of the time had a median survival of 46 months (about four years), as opposed to people treated for less than 90% of the time having a 15-month survival rate [24]. It has been established that it works by delaying the onset of stage 4 ALS rather than delaying the illness's progression or earlier stages [25]. When riluzole was first discovered to be an effective treatment for ALS, its usage in different healthcare systems was debatable due to the perception that the drug's cost was expensive and the survival benefit was insignificant. Its widespread adoption was prompted by a combination of health-economic analyses and patient advocacy pressure, though clearance took longer in other countries. Riluzole was authorized for treating ALS in the United Kingdom by the National Institute for Health and Care Excellence after a thorough cost-benefit study that considered the idea of quality-adjusted life years [25]. Nurtec, a new riluzole formulation absorbed by placing it under the tongue, is being developed because ALS frequently inhibits a patient's swallowing capacity. Troriluzole, a different prodrug form of riluzole, might be more easily absorbed by the body and have fewer side effects. For patients with various kinds of spinocerebellar ataxia (SCA), tririluzole is now being tested in phase three of clinical trials. By November 30, 2021, the experiment is anticipated to be finished, and it will shed further light on how well tririluzole works for SCA patients [26]. Hence we reach the result that 100 mg of riluzole once a day is judiciously safe and likely extends ALS patients' lives by roughly two months. More research is required to see how it affects older (over 75) individuals with more severe diseases [22].

Edaravone

The approval of edaravone was based on a study which included 137 ALS patients who were randomly randomised to receive 60mg of edaravone or a placebo during a 24-week treatment phase [27]. For the efficacy analysis, 66 patients received 60 mg of edaravone intravenously, while 68 received a placebo. It was done over six months in six cycles (four weeks on, two weeks off). Both groups of patients reported adverse events in 84% of cases, with patients using edaravone reporting serious events in 16% of cases and patients taking placebo in 24% of cases. One patient receiving edaravone and four receiving placebo had to be removed from the study due to adverse events [28]. When someone with ALS takes edaravone, it reduces the effects of oxidative stress, which may be connected to the death of motor neurons (nerve cells). Maintaining the health of motor neurons may aid muscle function [29]. It has been shown that this powerful free radical scavenger prevents tyrosine nitration in cerebrospinal fluid and improves motor function in mice models of ALS. The product is patented, and the FDA hasn't given the go-ahead for any generic edaravone versions [30].

Mitsubishi Tanabe Pharma Corporation, Osaka, Japan, created the edaravone clinical development programme with two main objectives: creating an efficient ALS treatment and assisting in creating clinical trial designs that would promote ALS research in the future [21]. Fifty ALS patients were treated with edaravone at a tertiary medical facility with an ALS speciality. Thirty patients finished a six-cycle administration, and sixteen had agreed to continue for extended treatment (10 men, six women; nine of them were limb-onset, seven were bulbar-onset; mean age calculated as 58.3 ± 10.3 years). The 34 drop-out patients in this included 24 who left because they believed the therapy was ineffective, five who passed away, and five whose whereabouts are unknown owing to loss of follow-up [31].

Edaravone is available as an infusion solution with thirty mg/100 ml. It is given via intravenous route as sixty mg infusions over sixty minutes every day for a total of 10 days out of a possible 14, which is further followed by a drug-free period of 14 days. Fourteen days of edaravone administration occur in the first month of treatment [32]. We should study the effects of edaravone further to increase the survival of patients having ALS.

This study aimed to determine whether riluzole and edaravone have any therapeutic impact in the treatment of ALS because there exists a limited number of studies and reviews available, so it was difficult to determine whether riluzole or edaravone is superior for treatment and boosting patient survival rates.

Riluzole was the first medicine licenced for ALS, and it has been demonstrated to extend patients' lives by up to two years. On the other hand, edaravone is the most recently authorised medicine for ALS. In clinical trials, riluzole appears to extend life in the early stages of ALS, whereas edaravone only suppresses symptom development. Choosing between these two treatments necessitates considering both their efficacy in clinical studies and how they exert their effects [33]. In Japanese patients, the therapeutic benefits of edaravone have been explored. To date, only the US has approved edaravone. In clinical trials, riluzole appeared to extend life in the early stages of ALS, whereas edaravone only halts the progression of symptoms. When determining which of these two treatments to utilise, evaluating their clinical efficacy and how they achieve their effects is critical. The study found edaravone to be safe, efficacious, and well-tolerated. When administered intravenously, the medication also causes thrombophlebitis. There are new questions due to recent developments in the understanding of ALS. These patients frequently receive prescriptions for drugs to treat their depression, sleep disorders, exhaustion, muscle cramps and pain, constipation, excessive saliva and phlegm spasticity, and constipation. When administered under a physical therapist's direction, physical therapy encourages patients to engage in specific, low-impact activities like walking, swimming, or stationary cycling. This helps the patient fight weariness and despair while enhancing cardiovascular health and muscle strength.

Occupational therapists can help patients learn how to use everyday aids like wheelchairs, walkers, ramps, and braces that help patients maintain some level of mobility and activity [34]. Unfortunately, the initial excitement for transgenic mouse models of the disease has not been quickly matched by improvements in treatment or prevention. Monogenic models may have unintentionally concealed how complex the human condition is; ALS has developed into a multisystem illness with various upstream aetiological streams leading to a final common pathway. However, ALS's clinical core is just as evident now as it was to Charcot and others. In light of the growing molecular complexity of ALS, we emphasize the continued importance of clinical observations.

## Conclusions

This review concludes that more research on edaravone's effectiveness in ALS is needed. Although there was a significant change in functional rating scores in two studies, one of these studies was small, open-label research. Thus we cannot rule out the possibility of a placebo effect. The participants in the other two trials did not represent a larger community of ALS patients due to the rigorous inclusion criteria, and the further two investigations did not demonstrate a statistically significant difference. Motor neuron illnesses are uncommon and rare, yet they produce severe impairment and a high mortality rate. The regional variation of the incidence of motor neuron disorders is not explained by any risk factors, implying that other unmeasured risk factors may play a role. The estimates offered here will be useful in planning services for people with motor neuron disorders around the world.

According to the data, edaravone appears to impact a specific subset of ALS patients in the early stage of the disease. In three of the four investigations, riluzole significantly reduced the chance of mortality or tracheostomy. Several possible therapeutic targets have been found since the invention of DNA technology. A greater understanding of ALS and therapeutics will result from medical advances and voluntary registration of persons with ALS in registries. Patients should be referred to a multidisciplinary team in the meantime to help them, their families, and carers manage the disease. Therefore, further research and studies are required for the treatment of ALS to improve the lives of patients, for having suitable treatment modalities for the patients and for treating patients accordingly.
